# Investigating the reliability and sex differences of digit lengths, ratios, and hand measures in infants

**DOI:** 10.1038/s41598-021-89590-w

**Published:** 2021-05-26

**Authors:** Luisa Ernsten, Lisa M. Körner, Martin Heil, Gareth Richards, Nora K. Schaal

**Affiliations:** 1 Department of Experimental Psychology, Heinrich-Heine-University, Universitätsstraße 1, 40225 Düsseldorf, Germany; 2 School of Psychology, Faculty of Medical Sciences, Newcastle University, Newcastle upon Tyne, UK

**Keywords:** Psychology, Biomarkers

## Abstract

Hands and digits tend to be sexually dimorphic and may reflect prenatal androgen exposure. In the past years, the literature introduced several hand and digit measures, but there is a lack of studies in prepubertal cohorts. The available literature reports more heterogeneous findings in prepubertal compared to postpubertal cohorts. The comparability of the available studies is further limited by the study design and different measurement techniques. The present study compared the reliability and sex differences of available hand and digit measures, namely digit lengths of 2D, 3D, 4D, 5D, digit ratios 2D:4D, 2D:5D, 3D:4D, 3D:5D, 4D:5D, relative digit lengths rel2, rel3, rel4, rel5, directional asymmetry of right and left 2D:4D (D_r-l_), hand width, length, and index of 399 male and 364 female 6-month-old German infants within one study using only indirect and computer-assisted measurements. The inter-examiner reliability was excellent while the test-retest reliability of hand scans was only moderate to high. Boys exhibited longer digits as well as wider and longer hands than girls, but smaller digit ratios, with ratios comprising the fifth digit revealing the largest effect sizes. Other hand and digit ratios revealed sex differences to some extent. The findings promote the assumption of sexual dimorphic hand and digit measures. However, by comparing the results of the available literature, there remains an uncertainty regarding the underlying hypothesis. Specifically in prepubertal cohorts, i.e. before the influence of fluctuating hormones, significant effects should be expected. It seems like other factors than the influence of prenatal androgens contribute to the sexual dimorphism in hand and digit lengths.

## Introduction

Sexual determination and differentiation can be observed in males and females of sexually reproducing species. Its origins lie in complex relations of biology, genetics, and social as well as physical environments^1,2^. While genetics primarily determine the gonads in the offspring, sex hormones like androgens and estrogens secondarily promote the phenotypic differentiation of males and females^3^. One of those phenotypic differences between male and female humans is the hand, as males exhibit generally bigger hands compared to females^4,5^.

In the past years, research suggested that hands and digits may serve as an indicator for sexual differentiation, as it is hypothesized that they are associated to the HOX genes^6^, which promote the development of the urogenital tract and external genitalia as well as the limb development^7,8^, and reflect differences in prenatal androgen exposure^9^. Therefore, there is extensive research on different hand and digit measures that are hypothesized to reflect different influences of prenatal androgens^10^. However, most studies regarding hand and digit measures are carried out with adult samples^11,12^, albeit the investigation of those ratios in young cohorts before onset of puberty, i.e. the effect of fluctuating hormones, seems intriguing as one can assume that sex and gender differences in this period are mainly associated to organizational effects of prenatal sex determining and differentiating factors such as genetics and sex hormones^13–15^. The literature and reported effects in hand and digit measures used as a marker for prenatal sex hormone exposure seem to be more homogeneous in adult samples^11^, while in prepubertal children there is considerable heterogeneity and age-dependent fluctuation regarding robust effects^16,17^. Yet, findings regarding different digit or hand measures as markers of prenatal androgens in younger cohorts are sparse.

Different hand and digit measures were discussed in the past research and shall be briefly presented (for an overview see supplementary table 1). The most discussed indicator for prenatal androgen exposure is the second to fourth digit ratio (2D:4D) that has also been associated with an amount of behavioral outcomes that are known to differ between males and females^18^. By comparing the length of the second digit to the length of the fourth digit, the general assumption of a lower 2D:4D in males compared to females has been revealed in many studies^11,17,19^. A sex difference in 2D:4D can be found as early as 14 weeks of gestational age^19^ but its stability over time varies across different studies^16,20^. Besides 2D:4D, the literature aimed to evaluate other hand and digit measures in humans as possible alternative markers for prenatal androgen exposure^21^. By computing digit ratios with every possible digit (excluding the thumb), i.e. the second (2D), third (3D), fourth (4D), and fifth digit (5D), significant differences between male and female ratios in different age groups could be observed in various studies^22–26^. However, a study regarding directly measured digit ratios in children aged 2–18 years revealed relevant fluctuations and argues that digit ratios other than 2D:4D do not serve as reliable indicators of prenatal androgen exposure^12^. On the other hand, the studies of Dressler & Voracek (2011) and Kumar et al. (2017) reported only small^26^ or no effects in 2D:4D^22^ and suggested that digit ratios with 5D as one of the components may reveal larger sex differences compared to 2D:4D^22^. But it is important to note that these studies used different measurement techniques. Whereas Kumar and colleagues (2017) measured the digit length dorsally^22^, most studies define the digit length over landmarks and flexion creases derived from the ventral surface of the hand^12,27^. Therefore, another study that used radiographs of children aged one month to 18 years and gives evidence for robust sex differences in different digit ratios^28^ might be difficult to compare to the aforementioned studies using digit lengths derived from measuring the soft tissue^12,22–26^. Another introduced measure is the relative digit length: By dividing the length of one digit by that of the sum of all four digits taken together, the contribution of each digit concerning a computed digit ratio can be determined^29^. It has been shown that there are sex differences in those relative digit lengths and that males in general exhibit larger relative lengths of 4D and 5D, while females exhibit larger relative lengths of 2D and 3D with low to medium effect sizes^29,30^. To the best of our knowledge, no study has considered the relative digit lengths in prepubertal cohorts. Another aspect to consider when looking at sexual dimorphism of the hand is that authors postulate that prenatal androgens have a different influence on the right versus left side of the body and digit ratios may differ in the right versus left hand^11,31,32^. In order to examine this assumption, it was Manning (2002) who introduced a measure reflecting directional asymmetry by subtracting left 2D:4D from right 2D:4D (D_r–l_ )^32^. Values < 0 indicate a right-biased asymmetry (i.e. 2D:4D is lower in the right relative to the left hand) which reflects high levels of early androgen exposure^33^. However, relatively little research has considered this variable in terms of sexual dimorphism and D_r-l_ in newborns remained uncorrelated towards testosterone levels in amniotic fluid and showed no significant sex difference^34^.

The hand itself as a discriminator between the sexes is widely used in forensic contexts to determine the sex of dismembered human extremities^35^. It could be shown that males tend to have larger and wider hands compared to females and also the hand index differs between the sexes^35–37^. Sex differences in hands are relatively stable^5^, however, in the fetal age no significant sex differences in hand length, width, and index [i.e. (Hand width/Hand length)*100] could be observed in radiographs of 50 fetuses between 20 and 40 weeks of gestational age^38^ and also in prepubertal children a considerable overlap between males and females considering the size of the hand remains^39^. With onset of the puberty, sex differences can be reliably observed^5^. A differentiation between males and females on the basis of hand measures may be more difficult in younger, i.e. prepubertal, cohorts compared to adolescent or adult samples. Albeit one can assume that the development of the hands relies on the same mechanisms as of digits, to the best of our knowledge, no study has investigated hand width, length, or index in the context of prenatal androgen exposure.

Despite considerable evidence for sexual dimorphic hand and digit measures, it is to note that studies differ in terms of their study design and measurement method. There are differences concerning the dimension of the hand from which lengths are examined (ventral^20,26,29^ versus dorsal^22,36^), the measurement technique (direct^17,24,30,36,37^ versus indirect^16,17,20,25,29^) and the used tools for measurement (computer based^25,29^ versus caliper^16,17,22,24,26,30,36,37^ versus ruler^17,19^) as well as the person examining the lengths (examiner^16,17,19,20,22,30^ versus self-measured^17,29^), or even the source for computing digit lengths, i.e. using radiographs^19,21^ rather than soft tissue^16,17,20,22,24–26,29,30,37^(see supplementary table 1). Comparing these different measurement techniques, they differ in terms of precision and can produce considerable variability in examinations and statistical effects^35,40,41^.

In sum, the literature provides valuable information about sex differences in hand and digit measures and it is hypothesized that the sexual dimorphic growth pattern is associated to the influence of genetics and prenatal androgen action. However, there are two main concerns regarding the available literature: (1) it appears that there is a general lack of studies examining and comparing different hand and digit ratios in younger, specifically in prepubertal cohorts. This is especially relevant as hand and digit growth appears to underlie age-dependent variations and a robust sex difference emerges with onset of puberty. And (2) the comparability of different studies is limited due to different measurement techniques used to assess hand and digit measures. The current study aims to compare hand and digit measures which have been already introduced in the literature, namely digit lengths of 2D, 3D, 4D, 5D, digit ratios of 2D:4D, 2D:5D, 3D:4D, 3D:5D, 4D:5D, relative digit lengths of 2D, 3D, 4D, 5D, as well as hand width, length, and index in a sample of infants (*N* = 763) within one study using the same measurement technique for each measure. We solely rely on indirect measurements using hand-scans and a computer-program as it is proposed that this technique shows the highest precision^40,42^. Based on previous studies, a sex difference between digit lengths, 2D:4D and other digit ratios is expected, and it is assumed that boys exhibit larger digit lengths but smaller digit ratios than girls. Furthermore, relative digit lengths, directional asymmetry in right and left 2D:4D (D_r–l_ ), and the hand width, length, and index are also investigated. Furthermore, we aim to investigate the reliability of repeated measurements and inter-examiner reliability to give valuable information on methodological considerations in examining hand and digit measures in very young cohorts.

## Methods

### Participants

Families with newborn children were recruited as part of other studies conducted between 2013 and 2018 at the Department of Experimental Psychology at Heinrich-Heine-University Düsseldorf. All parents spoke German fluently and almost all infants were White and from middle-class backgrounds. They were invited to take part in infant studies when their child was 6 months of age. In total 1381 (702 boys and 679 girls) infants with a mean age of 195.18 days (*SD* = 8.40) participated.

### Procedure

Families with 6-month-old infants came to the Department of Experimental Psychology at Heinrich-Heine-University Düsseldorf in order to take part in a mental rotation experiment [see^43^]. Informed consent was obtained from all parents and/or legal guardians of participating infants. After performing the mental rotation task, examiners took hand scans from a total of 1381 infants by pushing the ventral surface of the infant’s hand lightly onto the scanner glass and covering it up with a towel. Due to unexpected movements of the infants, particular digits could not be measured correctly. For data analysis, only hand scans with measurable 2D, 3D, 4D, and 5D were used (*N* = 790). In addition, outliers > 2 interquartile range (IQR) from the median were excluded (*N* = 27) resulting in a final sample of 763 infants/hand scans. The sample consisted of 399 boys and 364 girls. Of this sample, 180 scans (56% male) could be used for analysis of the hand length, width and index. A majority of the scans provided no valid measurement of hand length and width because the landmarks for those measures were covered by clothing or the thumb. Furthermore, for a subsample of 130 children (61% male), that were obtained as the last cohorts, scans were taken twice, i.e. at the beginning as well as at the end of the study to test for reliability of the hand scan measures. The families received a refund of their travel expenses, and the study was approved by the ethics committee of the Heinrich-Heine-University Düsseldorf in Germany.

### Measures

**Figure 1:**
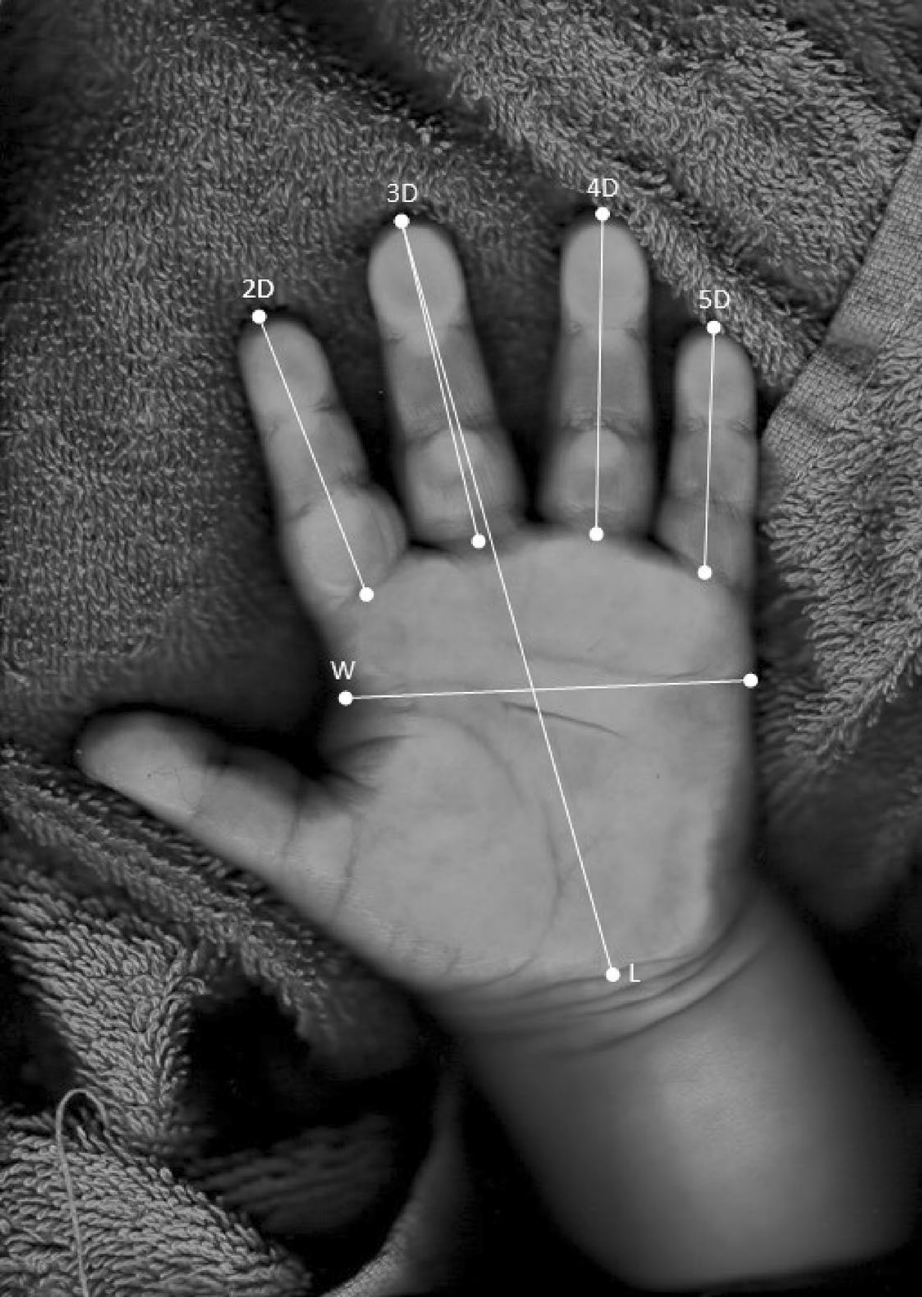
Example measurement of digit lengths (2D, 3D, 4D, 5D) and hand width (W) and length (L) using the freeware program *AutoMetric*^44^.

Scans of both right and left hand were obtained by an examiner using a FUJITSU fi-60F image scanner and digit lengths were measured using the freeware program *AutoMetric*^44^. Digital scans can be uploaded in the program and for each digit, excluding the thumb, the length can be determined by first clicking at the tip of each digit and second clicking at the midpoint of the ventral proximal flexion crease of each digit (see Fig. 1). The program then automatically computes the digit length into pixels. A monitor with 100 dpi was used, which means 100 pixels relate to 2.54 cm. The program was also used for measuring the hand width and length (see Table 1 and Fig. 1). All digit measures, i.e. ratios, relative digit lengths, the difference between right and left 2D:4D, and hand index, were derived from lengths measured in pixels and calculated using the statistical software *SPSS*^45^ (see Table 1). As shown for 2D:4D by Ribeiro, Neave, Morais and Manning (2016)^46^, indirect measures like hand scans produce larger sex differences and a higher measurement precision than direct measures; moreover, *AutoMetric* shows a high reliability for digit measurements and is superior to other computer-based measurement techniques^27,40^. Two independent examiners measured the hand scans and were blind to the sex of the children. Table 1 lists all hand and digit dimensions used and their computation base.

**Table 1:**
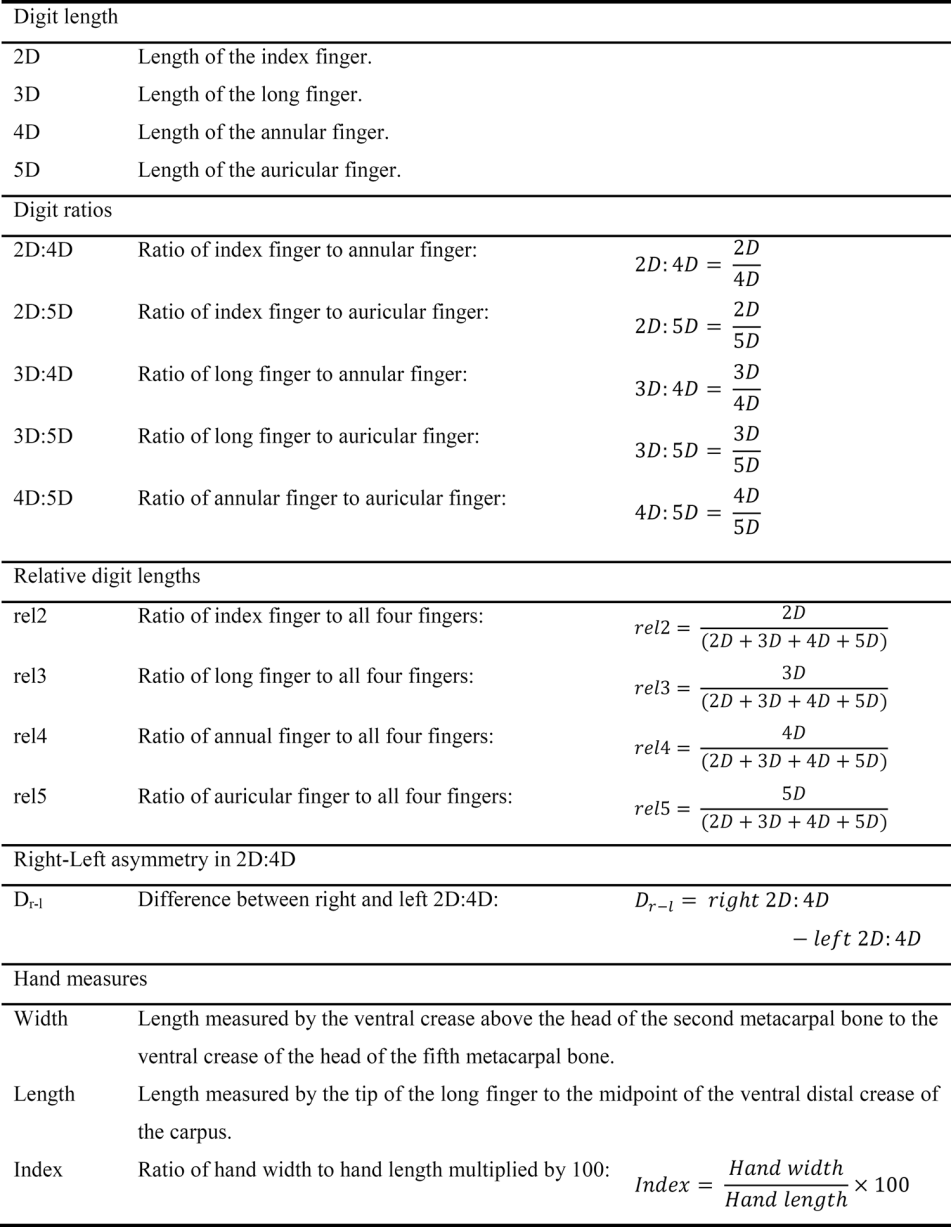
Overview of calculated digit and hand measures.

### Statistical analysis

For the reliability analysis of the measurements, the inter-examiner reliability is estimated by intra-class correlations (ICC) between the two independent examiners of the hand scans. The ICC is interpreted as follows: ICC < 0.50 means poor, 0.50 < ICC < 0.75 means fair, 0.75 < ICC < 0.90 means good, and ICC > 0.90 means excellent inter-examiner reliability^47^. Furthermore, for the subsample with additional hand scans taken before the experiment, the reliability of the measurements of hand scans taken before and at the end of the experiment (hereinafter referred to as test-retest-reliability) is estimated via Pearson correlations for every measure on both hands and averaged over both hands separated for boys, girls, and averaged over both sexes. Pearson correlations were interpreted as follows: low correlation *r* ≥ 0.10, moderate correlation* r* ≥ 0.30, and high correlation *r* ≥ 0.50^48^.

To test for main effects and interactions between the factors *sex* (male vs. female), *hand* (right vs. left), and *digit measure* (see Table 1 that lists different digit and hand measures/ factors and the factor levels), three 2 ×  2 ×  *n* (*n* for different factor levels) mixed ANOVAs with the between-subjects factor *sex*, and the within-subject factors *hand* and *digit measure* were conducted for digit length, digit ratio and relative digit length. In order to further disentangle the effects for sex, 2 × *n* mixed ANOVAs were computed separately for each hand with the between-subjects factor *sex* and the within-subject factor *digit measure*. For the hand width, length, and index, three 2 × 2 mixed ANOVAs with the within-subject factor *hand* and the between-subjects factor *sex* were conducted. To further investigate which digit measure was influenced by sex, post-hoc independent samples *t*-tests between boys and girls for each digit, digit ratio and hand measure were computed. The focus of the analyses is on sex differences. We report the results of the ANOVA for the sake of completeness, however, only the main effects of the factor sex are further investigated. The sex difference in right-left asymmetry in 2D:4D (D_r-l_) was tested using independent samples *t*-test. Multiple corrections were not applied, however, we report effects sizes *d* and their confident intervals as we believe that these measures promote the validity of our results.

Alpha-levels were set to 0.05 for each analysis, α ≤ 0.10 was interpreted as a statistical trend. Effect sizes are reported as *η*^*2*^_*p*_ and interpreted as *η*^*2*^_*p*_ ≥ 0.01 small effect, *η*^*2*^_*p*_ ≥ 0.06 medium effect, and *η*^*2*^_*p*_ ≥ 0.14 large effect^48^, or were converted to Cohen’s *d* and interpreted according to Cohen^48^— small effect *d* ≥ 0.20, medium effect *d* ≥ 0.50, and large effect *d* ≥ 0.80. *Greenhouse Geisser* adjustment was used to correct for violations of sphericity. All analyses were performed with *SPSS* version 27.0.

### Ethics declaration

The research was conducted in accordance with the Declaration of Helsinki.

## Results

### Reliability analysis

Intra-class correlations for the different digit lengths varied between 0.94 and 0.97 and were highly significant (all *p* < 0.001). Intra-class correlations for the hand width and length varied between 0.86 and 0.96 and also were highly significant (all *p* < 0.001). In supplementary table 2 and supplementary table 3 the computed intra-class correlations as well as their confidence intervals can be seen. The two measurements were averaged for each digit and hand measure on both hands to increase reliability. The following analyses were conducted with the averaged ratings.

The test-retest reliabilities of digit lengths are presented in supplementary table 4. They were significant for each digit and can be considered moderate to high. The reliabilities for the calculated digit measures were lower compared to those for digit lengths but can be also considered moderate to high. Scans taken at the beginning of the experiment that should be used for analyzing the hand width, length, and index were only available for 19 children (for the same reason as described above under *Procedure*) so that a reliability analysis could not be performed.

### Digit lengths

**Figure 2:**
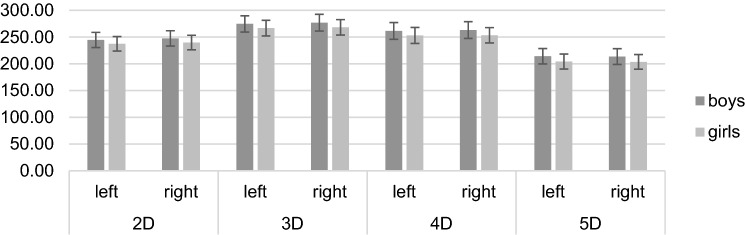
Means and standard deviations in right, left, and averaged digit lengths separately for boys and girls.

A 2 × 2 × 4 mixed ANOVA revealed significant main effects of the factor *hand*, *F* (1, 761) = 21.67, *p* < 0.001, *η*^*2*^_*p*_ = 0.03, the factor *digit measure*, *F* (2.30, 1,746.94) = 16,222.59, *p* < 0.001, *η*^*2*^_*p*_ = 0.96, and the factor *sex*, *F* (1, 761) = 86.28, *p* < 0.001, *η*^*2*^_*p*_ = 0.10, with digit lengths as the dependent variable. Furthermore, the digit measure*sex interaction, *F* (2.30, 1,746.94) = 6.41, *p* = 0.001, *η*^*2*^_*p*_ = 0.01, and the hand*digit measure interaction, *F* (2.28, 1,734.93) = 30.84, *p* < 0.001, *η*^*2*^_*p*_ = 0.04, were significant. The hand*sex and hand*digit measure*sex interactions were both non-significant (*p* ≥ 0.171). A 2 × 4 mixed ANOVA separately conducted for the right and left hand showed significant main effects of the factor *digit measure*, *F* (2.25, 1,710.06) = 11,892.09, *p* < 0.001, *η*^*2*^_*p*_ = 0.94, and the factor *sex*, *F* (1761) = 87.42, *p* < 0.001, *η*^*2*^_*p*_ = 0.10, on digit lengths and a significant digit measure*sex interaction, *F* (2.25, 1710.06) = 4.20, *p* = 0.012, *η*^*2*^_*p*_ = 0.01, for the right hand. For the left hand, there were significant main effects of the factor *digit measure*, *F* (2.37, 1803.36) = 11,991.80, *p* < 0.001, *η*^*2*^_*p*_ = 0.94, and the factor *sex*, *F* (1763) = 73.28, *p* < 0.001, *η*^*2*^_*p*_ = 0.62, on digit lengths and a significant digit measure*sex interaction, *F* (2.37, 1803.36) = 5.83, *p* = 0.002, *η*^*2*^_*p*_ = 0.01. Post-hoc *t*-tests revealed significant differences between girls and boys in every digit length (all *p* < 0.001, see Table 2) with girls having shorter digits than boys. The means and standard deviations are presented in Fig. 2.

**Table 2:**
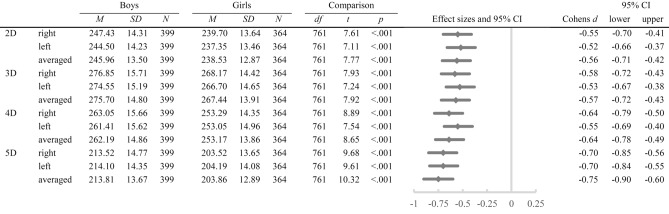
Means, standard deviations, post-hoc *t*-tests, and effect sizes for sex differences in digit lengths of both hands (N = 763).

### Digit ratios

**Figure 3:**
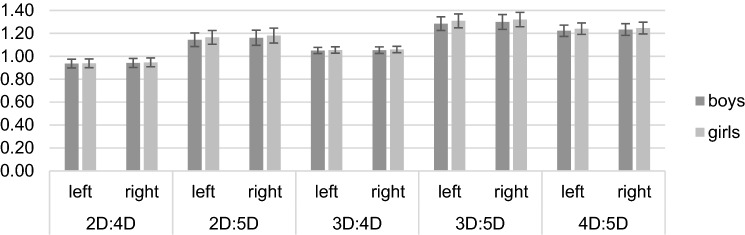
Means and standard deviations in right, left, and averaged digit ratios separately for boys and girls.

The 2 × 2   x 5 mixed ANOVA showed significant main effects of the factors *hand*, *F* (1, 761) = 44.77, *p* < 0.001, *η*^*2*^_*p*_ = 0.06, *digit measure*, *F* (1.95, 1481.67) = 16,493.65, *p* < 0.001, *η*^*2*^_*p*_ = 0.96, and *sex*, *F* (1761) = 31.32, *p* < 0.001, *η*^*2*^_*p*_ = 0.04, on digit ratios. Interactions were significant for digit measure*sex, *F* (1.95, 1481.67) = 14.39, *p* < 0.001, *η*^*2*^_*p*_ = 0.02, and for hand*digit measure, *F* (2.28, 1733.48) = 15.04, *p* < 0.001, *η*^*2*^_*p*_ = 0.02. The hand*sex and hand*digit measure*sex interactions were not significant (both *p* ≥ 0.166). Separate 2 × 5 mixed ANOVA of the right hand revealed significant main effects of *digit measure*, *F* (2.06, 1570.16) = 12,235.56, *p* < 0.001, *η*^*2*^_*p*_ = 0.94, and *sex*, *F* (1761) = 19.48, *p* < 0.001, *η*^*2*^_*p*_ = 0.03, and a significant digit measure*sex interaction, *F* (2.06, 1570.16) = 7.44, *p* = 0.001, *η*^*2*^_*p*_ = 0.01. Significant main effects of *digit measure*, *F* (2.00, 1520.49) = 12,345.43, *p* < 0.001, *η*^*2*^_*p*_ = 0.94, and *sex*, *F* (1761) = 27.21, *p* < 0.001, *η*^*2*^_*p*_ = 0.04, and a significant digit measure*sex interaction, *F* (2.00, 1520.49) = 15.07, *p* < 0.001, *η*^*2*^_*p*_ = 0.02, could also be observed for the left hand. Post-hoc *t-*tests revealed significant differences in every digit ratio between boys and girls (all *p* ≤ 0.004), except for right, left, and averaged 2D:4D as well as left 3D:4D, where no significant sex difference could be observed (see Table 3). In general, girls had larger digit ratios than boys. The means and standard deviations are presented in Fig. 3.

**Table 3:**
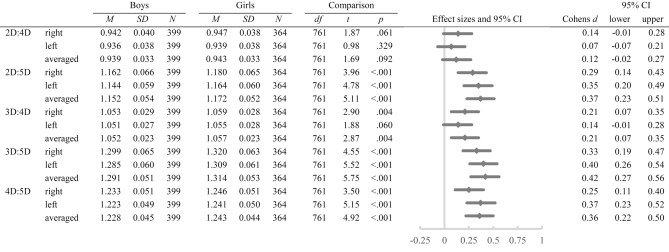
Means, standard deviations, post -hoc *t*- tests, and effect sizes for sex differences in digit ratios of both hands (*N* = 763).

### Relative digit lengths

**Figure 4:**
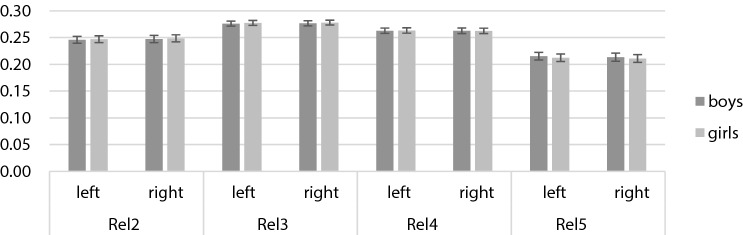
Means and standard deviations in right, left, and averaged relative digit lengths separately for boys, and girls.

The 2 × 2 × 4 mixed ANOVA revealed a significant main effect of the factor *digit measure*, *F* (2.25, 1710.31) = 16,600.99, *p* < 0.001, *η*^*2*^_*p*_ = 0.96, on relative digit lengths and significant digit measure*sex, *F* (2.25, 1710.31) = 18.57, *p* < 0.001, *η*^*2*^_*p*_ = 0.02, and hand*digit measure interactions, *F* (2.24, 1702.63) = 26.96, *p* < 0.001, *η*^*2*^_*p*_ = 0.03. The hand*digit measure*sex interaction was non-significant (*p* = 0.474). For the 2 × 4 mixed ANOVA separated by hand, a significant main effect of *digit measure*, *F* (2.19, 1665.92) = 12,085.74, *p* < 0.001, *η*^*2*^_*p*_ = 0.94, and a significant digit*sex interaction, *F* (2.19, 1665.92) = 11.70, *p* < 0.001, *η*^*2*^_*p*_ = 0.02, could be observed for the right hand. For the left hand, there was also a significant main effect of *digit measure*, *F* (2.30, 1750.94) = 11,999.04, *p* < 0.001, *η*^*2*^_*p*_ = 0.94, and a significant digit*sex interaction, *F* (2.30, 1750.94) = 17.33, *p* < 0.001, *η*^*2*^_*p*_ = 0.02. Post-hoc *t*-tests unveiled significantly larger rel2 (all *p* ≤ 0.020) and rel3 (all *p* ≤ 0.004) for girls compared to boys. rel4 did not significantly differ between girls and boys (all *p* ≥ 0.266). In rel5, boys exhibited significantly larger relative digit lengths than girls (all *p* < 0.001; see Table 4). The means and standard deviations are presented in Fig. 4.

**Table 4:**
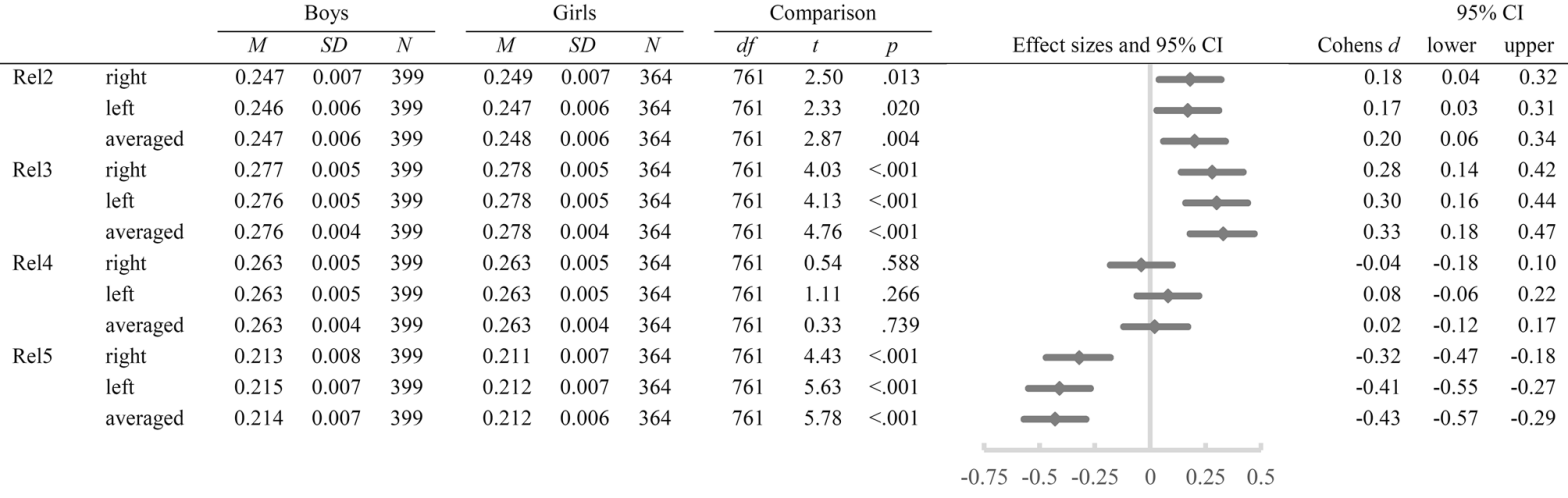
Means, standard deviations, post-hoc *t*-tests, and effect sizes for sex differences in relative digit lengths of both hands (N = 763).

### Right-left asymmetry in 2D:4D

The independent samples *t*-test showed no significant difference between girls’ (*M* = 0.01, *SD* = 0.04) and boys’ D_r-l_ (*M* = 0.01, *SD* = 0.04), *t* (761) = 0.88, *p* = 0.379, *d* = 0.06.

### Hand width, length, and index

**Figure 5:**
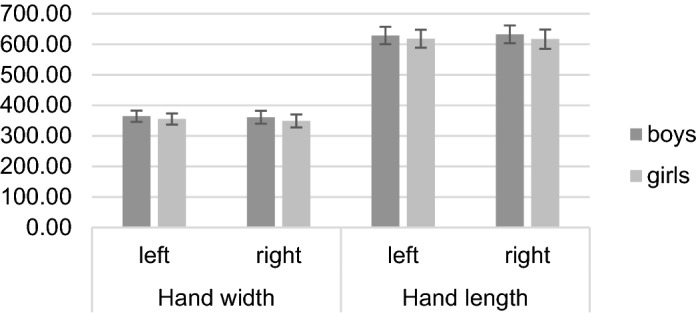
Means and standard deviations in right, left, and averaged hand width and length separately for boys and girls.

A 2 × 2 mixed ANOVA with hand width as the outcome variable revealed significant main effects of the factors *hand*, *F* (1, 178) = 16.42, *p* < 0.001, *η*^*2*^_*p*_ = 0.08, and *sex*, *F* (1, 178) = 15.34, *p* < 0.001, *η*^*2*^_*p*_ = 0.08. The interaction between these factors was non-significant (*p* = 0.186). Post-hoc *t*-tests revealed significant differences between boys and girls in the right, left and average over both hands’ width, with boys having wider hands than girls (all *p* ≤ 0.001; see Table 5). The means and standard deviations are presented in Fig. 5.

**Table 5:**
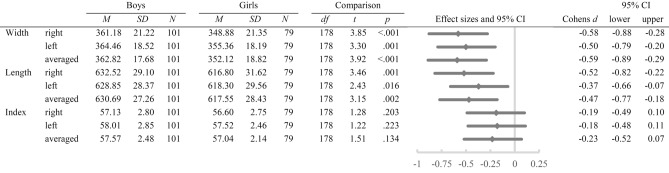
Means, standard deviations, post-hoc *t*-tests, and effect sizes for sex differences in hand width, length and index of both hands (*N* = 180).

For hand length, a 2 × 2 mixed ANOVA revealed a significant main effect of *sex*, *F* (1, 178) = 9.91, *p* = 0.002, *η*^*2*^_*p*_ = 0.05. The hand*sex interaction was marginally significant, *F* (1, 99) = 2.90, *p* = 0.091, *η*^*2*^_*p*_ = 0.02, while the main effect of *hand* was non-significant (*p* = 0.475). Post-hoc *t*-tests revealed significant sex differences in right, left and average over both hands’ lengths, with boys having longer hands than girls (all *p* ≤ 0.001; see Table 5). The means and standard deviations are presented in Fig. 5.

**Figure 6:**
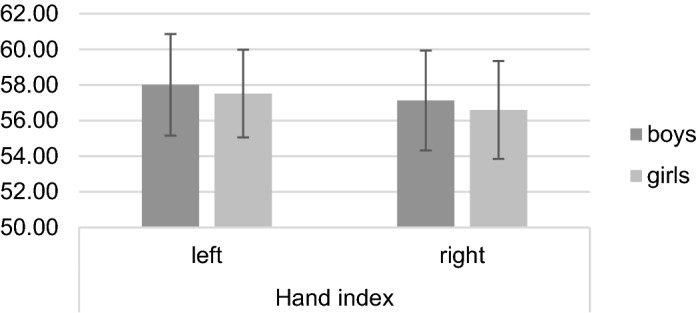
Means and standard deviations in right, left, and averaged hand index separately for boys and girls.

A 2 × 2 mixed ANOVA revealed a significant main effect of *hand*, *F* (1, 178) = 17.76, *p* < 0.001, *η*^*2*^_*p*_ = 0.09, on the hand index. The main effect of *sex* and the hand*sex interaction were non-significant (both *p* ≥ 0.145). Post-hoc *t*-tests showed no significant differences between boys’ and girls’ hand index (all *p* ≥ 0.134; see Table 5). The means and standard deviations are presented in Fig. 6.

## Discussion

Hand and digit measures are widely used to discriminate between the sexes and are hypothesized to reflect a different prenatal androgen exposure. In fact, there are robust sex differences in various measures, namely digit lengths and ratios, relative digit lengths as well as the hand width, length, and the hand index. However, those findings mostly rely on adult cohorts whereby findings in younger, specifically prepubertal cohorts remain more heterogeneous. Moreover, different measurement techniques impede the comparability of different studies as they result in considerable differences concerning measurement precision and possible bias. In the current study, we aimed to bridge the gap concerning the research of hand and digit measures as markers for prenatal androgen action in prepubertal cohorts by analyzing sex differences in a sample of 6-month-old infants and to give valuable methodological implications. Therefore, we analyzed the inter-examiner reliability as well as the test-retest reliability of digit length, hand width and length as well as the computed measures. Furthermore, we compared hand and digit measures which have been introduced in the literature, namely length of 2D, 3D, 4D, 5D, digit ratios 2D:4D, 2D:5D, 3D:4D, 3D:5D, 4D:5D, relative digit length rel2, rel3, rel4, rel5, directional asymmetry D_r-l_ of 2D:4D, as well as hand width, length, and the hand index within one study using the same measurement technique, i.e. indirect measurements and a computer program.

In the current study, the reliability analysis revealed excellent inter-examiner reliability while the test-retest reliability was only moderate to high. The results of this research provide supporting evidence for sex differences in digit lengths, ratios, and other digit and hand measures. Boys generally exhibited larger digits and bigger hands (i.e. hand width and length) with moderate to high effect sizes and smaller digit ratios compared to girls, a pattern that was evident for both right and left hands. Unexpectedly, the most commonly evaluated digit ratio, 2D:4D, showed no significant sex difference for left 2D:4D and only a marginally significant difference for the right and average of both hands with low effect sizes. For the relative digit lengths, low to moderate effect sizes and a different pattern of sex differences was observed for rel2 and rel3, with boys exhibiting smaller relative digit lengths than girls, whereas for rel5, they had larger relative digit lengths. There was no sex difference and low to moderate effects sizes in rel4, directional asymmetry of right and left 2D:4D (D_r-l_) and the computed hand index.

The reliability analysis confirmed that the inter-examiner reliability of digit and hand measures is nearly perfect, which is in line with other studies investigating indirect measurement techniques^27,40^ and indicates high reliability and repeatability of those indirect and computer-assisted measurement techniques. Regarding the test-retest reliability of different digit and hand measures, the results ranged from high to moderate. Only a few studies controlled multiple measurements among different examiners and found generally good test-retest reliabilities in adult cohorts^27,42^. Mikac, Buško, Sommer and Hildebrandt (2016) note that the reliability of repeated hand scans can be controlled by accurate instructions and a standardized measurement^27^, specifically by controlling the pressure with which hands are pressed onto the scanner glass, as this may lead to an error due to a shifting of important landmarks (e.g. flexion creases)^27^. These indications may not entirely apply to studies with young children because specifically very young children like infants cannot be adequately instructed and the examiner has to perform the hand scan. A further distortion by uncontrolled movements of the child and varying pressure applied during the scanning process may occur. Furthermore, because of the relatively higher amount of soft tissue in infants compared to adult cohorts^49^, varying pressure may lead to an even greater displacement of important landmarks. Regarding studies that have investigated hand and digit measures in very young cohorts, only one other study applied an indirect measurement technique using hand scans and also found generally low to moderate effect sizes^16^. It becomes apparent that in general more direct measurement techniques have been applied in very young cohorts (see supplementary table 1). Given the test-retest reliability in the current study, it may be indeed more applicable to directly measure hands and digits in infants, e.g. by using calipers or radiographs, as the indirect measurement of digit lengths and measures may account for solid differences in measurements.

However, due to the lack of studies investigating different measurement techniques within a sample, a comparison of different measurement techniques in infants is highly indicated. Furthermore, indirect measurements may be easier to implement compared to e.g. radiographs, and it becomes even more important to compare different measurement techniques within one study specifically in prepubertal, very young cohorts as well as to investigate the validity and reliability. Future studies should additionally investigate the test-retest reliability of measurement techniques and should not solely rely on the generally good inter- and intra-examiner reliabilities, especially in indirect measurements.

Regarding sex differences in different hand and digit measures, the results of the current study support the hypothesis that those measures can reliably differentiate between males and females as early as 6 months of age. The general assumption, that male hands and digits are larger than female whereby different digit ratios tend to be smaller in males compared to females, could also be supported^25,28^. However, these findings are contrasted by other studies, specifically in prepubertal cohorts, reporting considerable fluctuations in growth development as well as in digit measures^16,17,50^. Especially studies in prenatal or very young samples could not find sex differences in digit lengths and ratios from 9 to 40 weeks of gestational age^51^ as well as in newborns born between 37 and 40 weeks of gestational age^52^ or in hand length, width and index in deceased fetuses of 20 to 40 weeks of gestational age^38^. Studies investigating digit growth over a specific lifespan found larger digits in females from 2 years of age up to the age of 12 and a significant shift afterwards with males exhibiting longer digits after the age of 12^17,50^. This stands in direct contrast to our findings as we could replicate a male advantage in digit lengths that is reliably detected in adult cohorts. However, Gillam and colleagues (2008)^50^ as well as Manning and Fink (2018)^17^ did not examine the growth development between delivery and 2 years of age. The only available study reporting sex differences between 0 and 2 years of age, albeit just for 2D:4D, could find a significant difference but solely at 2 weeks of age with small to moderate effect sizes^16^. They also report significant age effects and weak correlations between measurements taken at different time points^16^. This is in fact a comparable result to the moderate test-retest reliability in our study that has been discussed in the previous paragraph. Regarding the growth of the hand, a similar pattern emerges by viewing the available literature, where robust sex differences can be found in adult samples, while younger cohorts show no significant sex differences in hand length, width and index (see supplementary table 1). Furthermore, sex differences in younger, prepubertal cohorts are described as more fluctuating and in adult cohorts as robust^5,53^. However, it is important to note that hand measures are mostly considered in forensic contexts and adult samples^35–37^, and in prepubertal cohorts most of the studies comparing hand length and width of girls and boys do that in terms of growth curves or prediction of body size in adult life^5,52,54^. There is a lack of studies considering hand length and width as markers for prenatal androgen exposure. In the current study, we included these measures as the influence of HOX genes and prenatal androgens, as stated in the introduction, refers to limb development and does not differentiate between the hand itself and individual digits^6–9^. The fact that malformations due to the expression of HOX genes can affect the hand itself as well as the digits, is another point that leads to the assumption of hand lengths and width as possible alternative markers^7,8^. Future research should investigate the biological basis of hand length and width regarding their ability as a reflection of the influence of HOX genes and prenatal androgens.

Considering digit ratios, the study of Knickmeyer and colleagues (2011) also shows no differences in 2D:4D in younger cohorts which is in line with our results^16^. This is in fact comparable to other findings that could not find sex differences in 2D:4D in younger cohorts^55–59^. This questions the assumption that 2D:4D is sexually dimorphic as early as the prenatal age^19,51^ and independent of age effects^17^. With regard to the other digit ratios, our results suggest stronger sex differences in ratios using 5D as one of their components, which has been already promoted by Kumar and colleagues (2017), albeit their measurement technique significantly differed from ours^22^. There are other studies reporting sex differences in other digit ratios, however, a comparison to our results is limited as these studies investigated only pubertal or postpubertal cohorts^23–26,29^. Another study by Manning (2012) investigated alternative digit ratios in prepubertal cohorts and reported a sexual dimorphic pattern in 2D:3D, 2D:4D, 2D:5D and right hands’ 3D:5D and 4D:5D, however, the author argues that these alternative ratios will not serve as good markers for sex differences since the age-dependent fluctuations are much more distinct compared to those in 2D:4D^12^. A general limitation of this assumption is the lack of longitudinal data^12^ that also accounts for our findings. A study investigating digit ratios in a sample of 108-7 to 13-year-olds and the age-related changes after 4 years found significant sex differences in left and right 2D:4D, left 2D:3D, left 2D:5D and right 3D:5D, but only for the first measurement, and a significant sex difference in left 2D:5D that was also apparent for the second measurement. After correcting for multiple testing, only the sex difference in 2D:3D remained significant. However, contrary to the assumption of Manning (2012)^12^, they have found a generally high stability for digit ratios between the two measurements. A study by McIntyre and colleagues (2005) analyzed the growth development between 1 month and 18 years of age and the sexual dimorphism of 2D, 3D, 4D as well as 2D:4D and 3D:4D and found significant differences in 3D:4D as early as the age of five^28^. Sex differences in 2D:4D occurred as early as 9 years of age. The authors conclude that digit ratios as markers for sex differences become more applicable with age, however, 3D:4D revealed sex differences in younger years more efficiently compared to 2D:4D^28^. There seems to be a general increase of sex differences in digit ratios with age and the literature supports the initial hypothesis that sex differences in prepubertal cohorts underlie significant age-related changes and thus findings remain more heterogeneous compared to adult respectively postpubertal cohorts.

As the discussed findings leave behind a considerable concern regarding the usefulness and applicability of hand and digit measures in prepubertal cohorts, it may be useful to mention the underlying hypothesis of these markers. It is proposed that the growth of hands and digits is influenced by prenatal androgen exposure. This could already be promoted by animal studies manipulating prenatal maternal and fetal hormone concentrations where a treatment with testosterone in pregnant Lewis rats led to a significant shortening of female and male offsprings’ 2D as well as male offsprings’ 4D^60^. The treatment had no effect on 2D:4D. In Wistar rats, a treatment with testosterone led to a shortening of the second digit and a simultaneous lengthening of the fourth digit in the offspring of pregnant rats compared to a control group that was treated with sesame oil^61^. In addition, the authors found a smaller 2D:4D in the testosterone group. In fact, Zheng & Cohn (2011) have shown that the growth of 2D and 4D in mice is controlled by the activity of androgen and estrogen receptors^62^. The authors showed that in general mice exhibit a similar pattern regarding 2D:4D with male mice having a smaller digit ratio than female mice. A deletion of the androgen receptor led to a larger 2D:4D ratio in males, while the deletion of the estrogen receptor led to a decrease of the 2D:4D ratio in males. Similarly, the inactivation of the androgen receptor led to a decrease in growth of the 4D while the inactivation of the estrogen receptor led to an increase. This growth pattern contributes to the sexual dimorphic pattern of the 2D:4D ratio in males and females. In general, a greater activity of androgen and estrogen receptors could be found in the 4D compared to the 2D^62^. A manipulation of hormonal concentrations during pregnancy is not possible in human studies due to ethical considerations. However, it is possible to compare the relative digit lengths in humans as proposed by Loehlin and colleagues (2009)^29^ and, with regard to Suchonova et al. (2019)^60^, Talarvičová et al. (2009)^61^ and Zheng & Cohn (2011)^62^, significant differences in the relative length of single digits are expected. In fact, we have found moderate to large differences in rel2 and rel3 with a female advantage, and a male advantage in rel5. Contrary to the assumption that 4D exhibits a greater activity of androgen and estrogen receptors^62^, we found no differences in rel4, although 4D is hypothesized to be the most sensitive for the effects of androgens and estrogens. In sum, the relatively high heterogeneity in different studies considering prepubertal cohorts discussed in previous paragraphs suggests that the initial hypothesis should be reinvestigated as the high stability of sex differences in adult or postpubertal cohorts and the reported increase of sex differences with age suggests other factors than prenatal androgen exposure that contribute to the sexual dimorphic pattern of hand and digit measures. If differences in digit lengths are primarily determined by the organizational influence of prenatal androgens and estrogens, a more stable sex difference in prepubertal cohorts should be expected.

It is well established that the human anatomy is lateralized and that specific bilateral structures are asymmetric^63^. This asymmetry in digits is hypothesized to rely on a different influence of prenatal androgens and can be measured as the directional asymmetry of right and left 2D:4D, D_r-l_^32^. However, D_r-l_ showed no significant difference between girls and boys of our sample, a finding that runs contrary to Manning et al. (2019)^64^ but is in accordance with other studies that failed to reveal such a difference^33,34^. In the current study, we did not further investigate the differences between the left and right hand as the focus of the present study was on sex differences in different hand and digit measures. However, except for hand length, a significant main effect for the factor *hand* could be revealed. But this effect, albeit descriptively, did not promote the general assumption that sex differences are more pronounced in the right hand^11^ as descriptive values were not always higher in the right hand (see Tables 2, 3, 4, and 5). A recent study showed that the asymmetry between the right and left hand in adults and children of 4 years of age is influenced by handedness, with right-handers exhibiting a more pronounced right-directional asymmetry compared to left-handers, thus emphasizing the influence of genetics on limb development^65^. In the current study, we did not control for handedness or other factors influencing the asymmetry and future studies should consider those alternative factors that may serve as explanations for differences in the right versus left hand. It may be additionally useful to compare D_r-l_ in digit ratios other than 2D:4D as, in regard to the general assumption of a different influence of prenatal androgens on the right versus left side of the body, this should be analogously observable in other digit ratios. As our study aimed to evaluate hand and digit measures that have already been cited in the available literature, this was beyond the scope of the current study. However, this is an interesting approach for future studies.

Although our overall results promote the general assumption of sexually dimorphic hands and digits as well as different hand and digit measures, our study deals with several limitations that may be important in the context of sexually dimorphic anthropometric measurements in very young cohorts. As the initial study and the subsequent study design did not primarily investigate sex differences in hand and digit measures but mental rotation^43^, the decision to examine a cohort of 6-month-olds was based on other theoretical considerations and hypotheses that did not focus on digit ratios as markers for prenatal androgen exposure. Furthermore, we acknowledge that while the results of the present sample regarding digit measures are based on a large sample of 763 infants, we could only measure hand width and length from 180 infants. Although a sample of 180 can be considered as a sufficient sample size, the missing data points need to be pointed out as a limitation of the study. Future studies should include in their study protocol a review of the hand scans so that non-evaluable scans can be identified early and rescanned which we unfortunately did not consider at the time the hand scans were taken. However, as the existing literature does not report many findings in cohorts between 0 and 2 years of age, our study gives important results to further compare different studies investigating sex differences in hand and digit ratios in very young cohorts. Furthermore, we could not control for the total body size as this variable was not evaluated. This may be additionally interesting as it would put the computed ratios in a more general context. Although we believe a strength of the present study is that we investigated the test-retest reliability, additional pre-scans were only available for a subset of our sample, as the initial procedure planned only the post-scans and the pre-scans were only implemented at the end of recruitment. Therefore, we had a limited number of available pre-scans. This was especially unfavorable for the test-retest reliability of hand width, length, and the hand index, as the few available pre-scans did not allow a meaningful analysis. Lastly, we did not correct for multiple testing. However, we reported the effect sizes and confidence intervals to promote the validity of our results.

In sum, our results provide supporting evidence for sex differences in different hand and digit measures as early as 6 months of age. However, the most prominent marker for the hypothesized relation between prenatal androgen exposure and the development of hands and digits, 2D:4D, did not reveal significant sex differences. Concerning the high heterogeneity in findings considering alternative digit ratios in prepubertal cohorts and the relatively high age-dependent fluctuation in digit growth comparing the more stable and generally larger sex differences in adult or postpubertal cohorts, there may be other factors that promote the sexually dimorphic pattern in hands and digits^66^. Kerrigan and Rogol (1992) argue, that the influence of sex hormones on growth development becomes more important with onset of puberty and that sex hormones have relevant, however complex and more secondary, moderating effects on the secretion of growth hormones^39^, which supports the general impression of more homogeneous findings with increasing age. Nevertheless, specifically 2D:4D reveals significant and interesting correlations with several human behaviors known to differ between males and females, e.g. play behavior in children^67–69^, as well as with several developmental disorders^70–72^, psychiatric disorders^73^ or even various types of cancer^74–79^. Similar results have been already reported for alternative digit ratios that could be linked to coronary heart diseases^80^, attention deficit hyperactivity disorder^81^, and externalizing and internalizing behaviors in children^82^. Although the underlying mechanisms, factors, and complex relations are not yet fully clarified, a valid and reliable measurement of hand and digit measures would be especially valuable in younger cohorts. Specifically for the early detection of disorders and diseases, it should be a major goal for future studies to further evaluate and provide for the quality of the hypothesized markers, i.e. hand and digit ratios. The current study provided important and valuable methodological implications and could support the assumptions of sex differences in different hand and digit measures as early as 6 months of age.

## Supplementary Information

Supplementary Information.

## Data Availability

The datasets analyzed in the current study are available in the Open Science Framework repository, https://osf.io/upzcx/?view_only=bf491c87e0ca4cab8ee62c7b0a841caf.
