# Supplementary material for: Investigating the reliability and sex differences of digit lengths, ratios, and hand measures in infants
Source: Sci Rep. 2021 May 26;11:10998. doi: 10.1038/s41598-021-89590-w (PMC8155043; doi:10.1038/s41598-021-89590-w)
Supplement: Supplementary file 1 — Supplementary Information. [file 41598_2021_89590_MOESM1_ESM.pdf]

Investigating the reliability and sex differences of digit lengths, ratios, and hand measures in infants

Luisa Ernsten, Lisa M. Körner, Martin Heil, Gareth Richards, & Nora K. Schaal

Supplementary table 1 Summary and overview of the literature (in alphabetical order) underlying our research question and highlighting the available hand and digit measures. No claim is made to completeness.

Study	N (f/m)	Age	Measurement technique	Investigated hand/ digit measure	Sex differences	Effect size for sex difference
Aboul-Hagag et al., 2011	250 females, 250 males	≥ 18 years	direct, anthropometer, sliding caliper, ventral	hand length	m > f	1.45[a, b, f]-1.47[a, b, e]
				hand width	m > f	2.42[a, b, f]-2.45[a, b, e]
				hand index	m > f	1.49[a, b, e]-1.50[a, b, f]
				2D	m > f	1.64[a, b, e]-1.70[a, b, f]
				4D	m > f	2.03[a, b, e]-2.09[a, b, f]
				2D:4D	m < f	1.46[a, b, e]-1.62[a, b, f]
Dressler & Voracek, 2011	75 females, 75 males	mean age 25.9 years (*SD* = 9.2)	indirect, scans, digital Vernier caliper, ventral	indirect:	indirect:	indirect:
				2D:3D	n.s.	0.04[b, e]-0.09[b, f]
				2D:4D	m < f	0.39[b, f]-0.40[b, e]
				2D:5D	m < f	0.57[b, e]-0.68[b, f]
			direct, digital Vernier caliper, ventral	3D:4D	m < f	0.42[b, f]-0.56[b, e]
				3D:5D	m < f	0.63[b, e]-0.68[b, f]
				4D:5D	m < f	0.43[b, e]-0.54[b, f]
				direct:	direct:	direct:
				2D:3D	n.s.	0.04[b, f]-0.20[b, e]
				2D:4D	n.s.	0.18[b, e]-0.24[b, f]
				2D:5D	m < f	0.33[b, e]-0.55[b, f]
				3D:4D	m < f	0.38[b, f]-0.47[b, e]
				3D:5D	m < f	0.52[b, e]-0.60[b, f]
				4D:5D	m < f[f]	0.26[b, e]-0.48[b, f]
Galis et al., 2010	158 females, 169 males	14-28 weeks of gestational age	indirect, radiography, ruler, ventral	2D:4D	m < f [g]	0.23[a, c]

Knickmeyer et al., 2011	183 females, 181 males	0-2 years	indirect, photocopies, Vernier calipers, ventral	2D:4D	m < f [g]	0.13-0.33
Krishan et al., 2011	100 females, 100 males	17-20 years	direct, sliding caliper, ventral and dorsal	hand length<br>hand width<br>hand index	m > f<br>m > f<br>m > f	1.69[a, b, f]-1.72[a, b, e]<br>2.05[a, b, f]-2.10[a, b, e]<br>0.43[a, b, f]-0.50[a, b, e]
Kumar et al., 2017	Study I: 53 females, 51 males	Study I: 16-48 years	direct, Vernier calipers, dorsal	Study I:<br>2D:3D<br>2D:4D<br>2D:5D<br>3D:4D<br>3D:5D<br>4D:5D	Study I:<br>n.s.<br>n.s.<br>m < f<br>n.s.<br>m < f<br>m < f	Study I:<br>0.06[b, f]-0.15[b, e]<br>0.00[b, f]-0.31[b, e]<br>0.36[b, e]-0.72[b, f]<br>0.06[b, f]-0.28[b, e]<br>0.48[b, e]-0.69[b, f]<br>0.68[b, e]-0.83[b, f]
	Study II: 68 females, 86 males	Study II: 17-28		Study II:<br>2D:3D<br>2D:4D<br>2D:5D<br>3D:4D<br>3D:5D<br>4D:5D	Study II:<br>n.s.<br>n.s.<br>m < f<br>n.s.<br>m < f<br>m < f	Study II:<br>0.06[b, d, f]-0.18[b, e]<br>0.00[b, f]-0.15[b, e]<br>0.42[b, e]-0.48[b, d]<br>0.00[b, f]-0.05[b, e]<br>0.37[b, e]-0.50[b, f]<br>0.38[b, e]-0.57[b, f]
	Study III: 33 females, 31 males	Study III: 3-7.6 years		Study III:<br>2D:3D<br>2D:4D<br>2D:5D<br>3D:4D<br>3D:5D<br>4D:5D	Study III:<br>n.s.<br>n.s.<br>n.s.<br>n.s.<br>m < f<br>m < f	Study III:<br>0.33[b, e, f]-0.39[b, d]<br>0.07[b, f]-0.16[b, e]<br>0.25[b, e]-0.53[b, f]<br>0.13[b, e]-0.29[b, d, f]<br>0.44[b, e]-0.70[b, f]<br>0.46[b, e]-0.63[b, d]
Kyriakidis, 2021	80 females, 80 males	19-91 years	direct, electronic Vernier caliper, ventral	2D<br>3D<br>4D<br>5D<br>2D:3D<br>2D:4D<br>2D:5D<br>3D:4D<br>3D:5D	m > f<br>m > f<br>m > f<br>m > f<br>m < f<br>m < f<br>m < f<br>m < f [f]<br>n.s.	

				4D:5D	m < f [e]	
Kyriakidis & Papaioannidou, 2008	60 females, 60 males	19-25 years	direct, electronic Vernier caliper, ventral	2D	m > f	1.23[a, b, f]-1.24[a, b, e]
				3D	m > f	1.56[a, b, e]-1.67[a, b, f]
				4D	m > f	1.53[a, b, f]-1.60[a, b, e]
				5D	m > f	1.38[a, b, f]-1.57[a, b, e]
				2D:3D	m < f	0.62[a, b, e]-0.67[a, b, f]
				2D:4D	m < f	0.63[a, b, f]-0.75[a, b, e]
				2D:5D	m < f	0.62[a, b, f]-0.72[a, b, e]
				3D:4D	m < f [e]	0.16[a, b, f]-0.36[a, b, e]
				3D:5D	m < f [e]	0.16[a, b, f]-0.57[a, b, e]
				4D:5D	m < f [e]	0.09[a, b, f]-0.35[a, b, e]
Loehlin et al., 2009	400 females, 300 males	2/3 10-16 years, 1/3 ≥17 years	indirect, scans, computer program	rel2	m < f	0.46[b, f]-0.66[b, e]
				rel3	m < f	0.09[b, f]-0.22[b, e]
				rel4	m > f	0.10[b, f]-0.18[b, e]
				rel5	m > f	0.29[b, f]-0.33[b, e]
				2D:3D	m < f	0.32[b, f]-0.42[b, e]
				2D:4D	m < f	0.36[b, f]-0.58[b, e]
				2D:5D	m < f	0.39[b, f]-0.50[b, e]
				3D:4D	m < f	0.14[b, f]-0.33[b, e]
				3D:5D	m < f	0.22[b, f]-0.27[b, e]
				4D.5D	m < f	0.13[b, e]-0.17[b, f]
Manning, 2002 (Manning et al., 2000)	661 females, 567 males	children and adults	indirect, scans, Vernier calipers, ventral<br>direct, Vernier calipers, ventral	D_{r-l}	n.s.	0.04[a, b]
Manning, 2012	340 females, 340 males	2-18 years	direct, Vernier calipers, ventral	2D:3D	m < f [g]	0.17[a, c, e]-0.30[a, c, f]
				2D:4D	m < f [g]	0.26[a, c, e]-0.30[a, c, f]
				2D:5D	m < f [g]	0.32[a, c, f]-0.35[a, c, e]
				3D:4D	n.s. [g]	0.03[a, c, f]- 0.15[a, c, e]
				3D:5D	m < f [e, g]	0.14[a, c, f]-0.27[a, c, e]
				4D:5D	m < f [e, g]	0.11[a, c, f]-0.17[a, c, e]
Manning & Fink, 2018	Sample I: 340 females, 340 males (40 per age group)	Sample I: 2-18 years	Sample I: direct, Vernier calipers, ventral	Sample I:	Sample I:	Sample I:
				2D	m > f [g]	0.20[a, c, e]
				4D	m > f [g]	0.38[a, c, e]

	 Sample II: 42,843 females, 46,402 males	 Sample II: 18-30 years	 Sample II:<br>self-measurement, ruler, ventral	2D:4D<br>Sample II:<br>2D<br>4D<br>2D:4D	m < f<br>Sample II:<br>m > f [g]<br>m > f [g]<br>m < f	0.26[a, c, e]<br>Sample II:<br>0.45[a, c, e]<br>0.49[a, c, e]<br>0.21[a, c, e]
McIntyre et al., 2005	56 females, 68 males	1 month-18 years	indirect, radiography, computer program	2D<br>3D<br>4D<br>2D:4D<br>3D:4D	m > f<br>m > f<br>m > f<br>m < f [g]<br>m < f [g]	 <br><br>0.35[a, c, f]<br>0.44[a, c, f]
McIntyre et al., 2006	537 females, 523 males	2-10 years	indirect, radiography, computer program	2D:4D<br>3D:4D	m < f<br>m < f	0.24[a, b, f]<br>0.61[a, b, f]
McFadden & Shubel, 2002	62 females, 60 males	heterosexual males *M* =19.0, heterosexual females *M* =19.1, homosexual males *M* =22.0, homosexual females *M* =20.7 years	indirect, scans, computer program	2D:3D<br>2D:4D<br>2D:5D<br>3D:4D<br>3D:5D<br>4D:5D	m < f<br>m < f<br>m < f<br>m < f<br>m < f<br>n.s.	0.49[b, f]-0.50[b, e]<br>0.74[b, f]-0.85[b, e]<br>0.68[b, f]-0.76[b, e]<br>0.54[b, f]-0.72[b, e]<br>0.39[b, f]-0.51[b, e]<br>0.13[b, e, f]
Raziye et al., 2016	27 females, 23 males	20-40 weeks of gestational age	indirect, mammography and radiography, digital caliper	2D<br>3D<br>4D<br>5D<br>2D:4D<br>hand length<br>hand width<br>hand index	n.s.<br>n.s<br>n.s<br>n.s<br>n.s<br>n.s<br>n.s.<br>n.s.	
Richards et al., 2019	54 females, 52 males	*median* = 27.18 h after birth	indirect, scans, Vernier calipers, ventral	2D:4D<br>D_{r-l}	m < f [f]<br>n.s.	0.21[a, b, e] – 0.57[a, b, f]<br>0.30[a, b]
Stenstrom et al., 2011	235 females, 178 males	17-44 years	direct, digital calipers, ventral	2D:4D<br>rel2	m < f<br>m < f	0.30[b, e]<br>0.31[b, e]

Wong & Hines, 2016	70 females, 56 males	three age groups: 20-26 months, 27-33 months, 34-40 months	indirect, scans, ventral, in pixel using a computer program	2D:4D	m < f	0.61[c]

Note: n.s. – not significant, [a] Computed from test statistic, [b] Comparison of group means, [c] Main effect of factor sex, [d] Mean of right and left hand, [e] right hand, [f] left hand, [g] age effects.

Supplementary table 2 Intra-class correlations and 95% confident intervals of digit length measurements of two independent examiners.

			ICC	95% CI	
				upper	lower
2D	pre	right	.97	.96	.98
		left	.96	.95	.97
	post	right	.96	.95	.97
		left	.95	.95	.96
3D	pre	right	.97	.96	.98
		left	.97	.96	.98
	post	right	.97	.96	.97
		left	.95	.95	.96
4D	pre	right	.96	.95	.98
		left	.97	.97	.98
	post	right	.96	.96	.97
		left	.94	.93	.95
5D	pre	right	.97	.95	.98
		left	.95	.93	.97
	post	right	.96	.95	.96
		left	.95	.94	.95

Supplementary table 3 Intra-class correlations and 95% confident intervals of hand length and width measurements of two independent examiners.

			ICC	95% CI	
				upper	lower
Hand length	pre	right	.96	.93	.97
		left	.94	.89	.96
	post	right	.95	.93	.96
		left	.93	.92	.95
Hand width	pre	right	.86	.78	.92
		left	.96	.92	.97
	post	right	.94	.93	.95
		left	.95	.94	.96

Supplementary table 4 Test-retest reliability of pre and post scans for boys, girls, and overall.

		Boys			Girls			Overall		
		r	*p*	*N*	*r*	*p*	*N*	*r*	*p*	*N*
2D	right	.77	<.001	66	.86	<.001	64	.81	<.001	130
	left	.78	<.001	66	.84	<.001	64	.81	<.001	130
	averaged	.84	<.001	66	.92	<.001	64	.88	<.001	130
3D	right	.81	<.001	66	.90	<.001	64	.85	<.001	130
	left	.83	<.001	66	.87	<.001	64	.85	<.001	130
	averaged	.88	<.001	66	.94	<.001	64	.91	<.001	130
4D	right	.80	<.001	66	.72	<.001	64	.77	<.001	130
	left	.88	<.001	66	.84	<.001	64	.87	<.001	130
	averaged	.91	<.001	66	.86	<.001	64	.89	<.001	130
5D	right	.68	<.001	66	.74	<.001	64	.72	<.001	130
	left	.86	<.001	66	.82	<.001	64	.85	<.001	130
	averaged	.84	<.001	66	.88	<.001	64	.87	<.001	130
2D:4D	right	.58	<.001	66	.37	.002	64	.50	<.001	130
	left	.55	<.001	66	.68	<.001	64	.63	<.001	130
	averaged	.65	<.001	66	.68	<.001	64	.67	<.001	130
2D:5D	right	.42	<.001	66	.54	<.001	64	.49	<.001	130
	left	.62	<.001	66	.59	<.001	64	.63	<.001	130
	averaged	.62	<.001	66	.67	<.001	64	.67	<.001	130
3D:4D	right	.62	<.001	66	.37	.003	64	.52	<.001	130
	left	.56	<.001	66	.50	<.001	64	.53	<.001	130
	averaged	.71	<.001	66	.56	<.001	64	.66	<.001	130
3D:5D	right	.49	<.001	66	.65	<.001	64	.59	<.001	130
	left	.69	<.001	66	.62	<.001	64	.67	<.001	130
	averaged	.69	<.001	66	.75	<.001	64	.74	<.001	130
4D:5D	right	.54	<.001	66	.70	<.001	64	.64	<.001	130
	left	.68	<.001	66	.65	<.001	64	.67	<.001	130
	averaged	.69	<.001	66	.80	<.001	64	.75	<.001	130
rel2	right	.46	<.001	66	.43	<.001	64	.46	<.001	130
	left	.56	<.001	66	.63	<.001	64	.61	<.001	130
	averaged	.60	<.001	66	.65	<.001	64	.64	<.001	130
rel3	right	.56	<.001	66	.62	<.001	64	.60	<.001	130
	left	.66	<.001	66	.55	<.001	64	.61	<.001	130
	averaged	.73	<.001	66	.72	<.001	64	.73	<.001	130
rel4	right	.70	<.001	66	.42	<.001	64	.57	<.001	130
	left	.54	<.001	66	.66	<.001	64	.61	<.001	130
	averaged	.70	<.001	66	.70	<.001	64	.71	<.001	130
rel5	right	.45	<.001	66	.65	<.001	64	.56	<.001	130
	left	.69	<.001	66	.61	<.001	64	.67	<.001	130
	averaged	.67	<.001	66	.74	<.001	64	.72	<.001	130
D_{r-l}		.40	.001	66	.33	.008	64	.36	<.001	130
